# Modeling Pediatric Body Mass Index and Neighborhood Environment at Different Spatial Scales

**DOI:** 10.3390/ijerph15030473

**Published:** 2018-03-08

**Authors:** Lauren P. Grant, Chris Gennings, Edmond P. Wickham, Derek Chapman, Shumei Sun, David C. Wheeler

**Affiliations:** 1 Department of Biostatistics, Virginia Commonwealth University, Richmond, VA 23298, USA; pacele@vcu.edu (L.P.G.); shumei.sun@vcuhealth.org (S.S.); 2 Department of Environmental Medicine and Public Health, Mount Sinai, New York, NY 10029, USA; chris.gennings@mssm.edu; 3 Children’s Hospital of Richmond, Virginia Commonwealth University, Richmond, VA 23298, USA; edmond.wickham@vcuhealth.org; 4 Department of Family Medicine and Population Health, Virginia Commonwealth University, Richmond, VA 23298, USA; derek.chapman@vcuhealth.org

**Keywords:** spatial scale, model selection, lasso, body mass index, obesity

## Abstract

In public health research, it has been well established that geographic location plays an important role in influencing health outcomes. In recent years, there has been an increased emphasis on the impact of neighborhood or contextual factors as potential risk factors for childhood obesity. Some neighborhood factors relevant to childhood obesity include access to food sources, access to recreational facilities, neighborhood safety, and socioeconomic status (SES) variables. It is common for neighborhood or area-level variables to be available at multiple spatial scales (SS) or geographic units, such as the census block group and census tract, and selection of the spatial scale for area-level variables can be considered as a model selection problem. In this paper, we model the variation in body mass index (BMI) in a study of pediatric patients of the Virginia Commonwealth University (VCU) Medical Center, while considering the selection of spatial scale for a set of neighborhood-level variables available at multiple spatial scales using four recently proposed spatial scale selection algorithms: SS forward stepwise regression, SS incremental forward stagewise regression, SS least angle regression (LARS), and SS lasso. For pediatric BMI, we found evidence of significant positive associations with visit age and black race at the individual level, percent Hispanic white at the census block group level, percent Hispanic black at the census tract level, and percent vacant housing at the census tract level. We also found significant negative associations with population density at the census tract level, median household income at the census tract level, percent renter at the census tract level, and exercise equipment expenditures at the census block group level. The SS algorithms selected covariates at different spatial scales, producing better goodness-of-fit in comparison to traditional models, where all area-level covariates were modeled at the same scale. These findings underscore the importance of considering spatial scale when performing model selection.

## 1. Introduction

In public health research, it has been well established that geographic location plays an important role in influencing health outcomes [1,2,3,4,5,6,7]. In recent years, there has been an increased emphasis on the impact of neighborhood or contextual factors as potential risk factors for overweight children or childhood obesity [8,9,10]. While individual-level factors such as diet and physical activity certainly play a part in a child’s risk for obesity, to ignore the importance of the neighborhood environment in which a child resides could be costly to the inference that a researcher makes. Davison and Birch [8] portrayed the levels of influence (individual, familial, and community) that surround a child’s weight status as a set of concentric rings around child weight status. Individual-level risk factors for childhood obesity are shown in the innermost ring, namely: a child’s dietary intake, physical activity, and sedentary behavior. The outer ring is for community or neighborhood-level factors. Examples of neighborhood factors include access to food sources, access to recreational facilities, and neighborhood safety. Socioeconomic status (SES) variables, such as housing tenure and median household income, are additional examples of neighborhood or area-level variables.

It is common for neighborhood or area-level variables to be available at multiple spatial scales (SS) or geographic units (e.g., census block group, census tract, ZIP code), and selection of the spatial scale for area-level variables can be considered a model selection problem. In the literature, various studies have been conducted to consider spatial scale when modeling associations between area-level variables and health outcomes [3,4,5,6,7]. Traditionally, area-based covariates in regression models are assumed to all be appropriately modeled at the same spatial scale, where the general rule of thumb is that smaller is better in terms of the chosen spatial unit [4,5,11]. However, some studies have shown that different area-level covariates can influence health outcomes at different spatial scales [3,6,7,12,13]. As an example, Root [6] finds that the strength of association between area-level SES covariates and orofacial cleft risk changes when using different spatial scales (i.e., 4000-m buffer, census tract, census block group), and that poverty and unemployment have a stronger relationship with risk for cleft palate at smaller and larger scales, respectively.

Given that different area-level covariates appear to have different effects at different scales, we seek an alternative to the conventional approach of choosing one spatial scale at which to model all area-level covariates. Rather, we opt to allow each area-level variable to enter a model at its optimal spatial scale. Grant et al. [14] presented four model selection algorithms that can be used for the selection of area-level covariates in regression models in order to ensure that the best spatial scale is selected for each area-level covariate. The objective of this paper is to model the variation in body mass index (BMI) in a study of pediatric patients at the Virginia Commonwealth University (VCU) Medical Center, while considering the selection of spatial scale for a set of neighborhood-level variables available at multiple spatial scales using the spatial scale selection algorithms.

## 2. Materials and Methods

### 2.1. Study Data

The patient data were composed of children from 2–17 years of age who visited the VCU Medical Center between September 2009 and December 2012. Patient data, which included demographic information in addition to residential addresses at the time of hospital visit, were accessed through the VCU Cerner Health System. Patient addresses were geocoded using Business Analyst Desktop 10.1 software (Environmental Systems Research Institute, Redlands, CA, USA), and linked to the United States (US) Census 2010 data (obtained from the US Census Bureau), StreetMap data from the Business Analyst Desktop 10.1, 2010 business data, 2011 consumer spending data, and 2013 crime index data (all obtained from ESRI) to assign various neighborhood-level socioeconomic and crime index covariates to each patient. We considered neighborhood-level variables at three different geographically defined units ordered from smallest to largest in area: the census block (CBK), census block group (CBG), and census tract (CT).

The outcome variable of interest was BMI z-score (BMIZ). Individual-level covariates included age, gender, race/ethnicity, and distance to the VCU Medical Center in miles. Area-level covariates included the following: population density; percent of population that is black, Hispanic white, and Hispanic black; median household income (MHI); percent of renter-occupied housing units; percent of vacant housing units; and crime indices. Crime indices included total crime, crimes against persons, and property crime. In addition, park density, restaurant density, and exercise equipment expenditures were considered as surrogates for green space, food access, and physical activity, respectively. All area-level variables were available at both the CBG and CT levels, with one variable (population density) also available at the CBK level. For further details on the variables, see Table 1.

**Figure 1:**
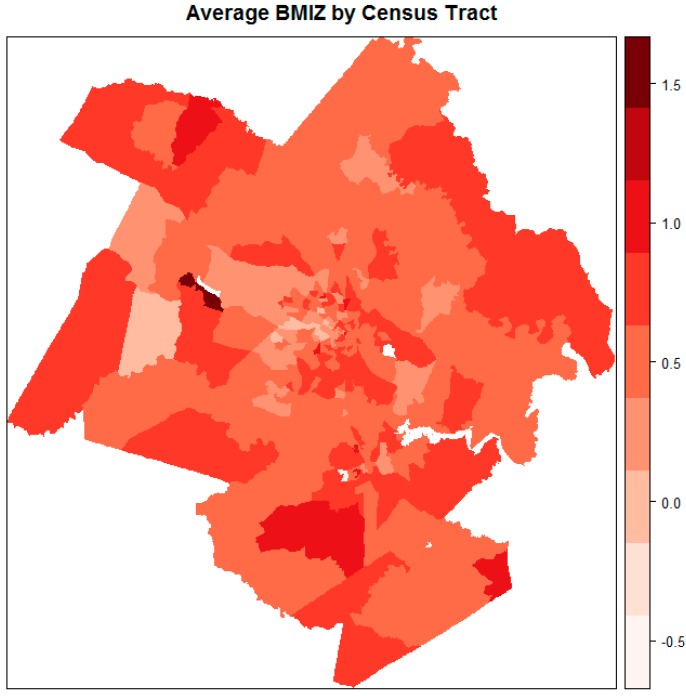
Average body mass index (BMI) z-score (BMIZ) by census tract among study patients in the Richmond metropolitan statistical area.

The majority of the patients (79%) resided in the Richmond metropolitan statistical area (MSA). A map of average BMIZ by census tract for the Richmond MSA, as delineated by the Office of Management and Budget (OMB) in December 2009 [15], is shown in Figure 1. It is evident that mean BMIZ tended to be higher for areas along the perimeter of the Richmond MSA.

Of the total 29,471 patient observations, 1933 observations were excluded for the following reasons. We removed clinically underweight patients (*n* = 1482), where underweight was defined as having a BMI z-score that was less than the fifth percentile [16]. The rationale for the exclusion of underweight children was twofold: (1) to remove extreme outliers from the data, and (2) in following the spirit of other studies, to remove the portion of the study population for which a BMI increase would actually be wanted [17]. We also excluded observations of patients who were non-white or non-black (*n* = 449) in order to limit the study population to black and white patients, and two observations due to missing values. Thus, the sample size used for analyses was *n* = 27,538.

### 2.2. Statistical Analysis

We modeled the BMI z-scores of pediatric patients using four spatial scale selection algorithms proposed by Grant et al. [14]: SS forward stepwise regression, SS incremental forward stagewise regression, SS least angle regression (LARS), and SS lasso. These SS algorithms selectively choose the best spatial scale at which to model each area-level covariate. Applying the SS algorithms produced baseline SS main effects models. For the LARS and lasso algorithms, we chose the model that had the minimum Akaike’s information criterion (AIC). For fitting the SS forward stepwise model, we set the stopping criterion ε = 1 based on a substantial difference in AIC being 4 to 7 [18]. For the SS forward stagewise algorithm, we set the step size at 0.001, and the tolerance at 0.01. The significance level for these analyses was α = 0.05. In order to acquire approximate *p*-values and AIC measures, we used the covariates that were selected by each of the SS stagewise, SS LARS, and SS lasso models to fit ordinary least squares (OLS) regression models for BMI z-scores. All of the analyses were conducted in the R computing environment [19]. The four spatial scale selection algorithms were implemented in an R package, spselect [20]. The code for setting up the data and running the algorithms is included in Appendix A.

Due to the hierarchical nature of the BMI dataset, we considered the inclusion of random effects (RE) at different spatial scales in an effort to account for the potentially correlated nature of subjects within a particular spatial scale. For each of the SS algorithms, we took the model-selected covariates and fit three linear mixed-effects models [21], namely, random intercept models with an RE at the CBG, an RE at the CT, and REs at both the CBG and CT. We obtained maximum likelihood (ML) estimates of the fixed effects and corresponding *p*-values [22]. We also mapped the random effects for the Richmond MSA.

As an additional analysis step, we fitted linear models with interaction terms. We fit OLS regression models using the covariates that were selected by each of the respective SS algorithms, and any covariates deemed to be of biological importance, along with interaction terms of interest. Nine interaction terms based on clinical recommendation were included. Interactions between male and the following covariates were examined: population density, MHI, park density, and exercise equipment. Similarly, interactions between black and the aforementioned covariates were also examined, in addition to an interaction between the distance to the VCU Medical Center and MHI. As described for the SS main effects models, we fit these interaction models with random effects and obtained ML estimates.

For the SS algorithms, we standardized both the outcome and predictor variables for the purposes of removing the intercept term, particularly for the lasso algorithm. For models fit with interaction terms, we left the outcome variable BMIZ unstandardized, and standardized all of the predictor variables according to Gelman’s [23] scaling recommendations, where dummy variables were centered, and continuous predictor variables were standardized by centering and dividing by two standard deviations rather than one. In this way, regression coefficient estimates, particularly those for dummy variables and interaction terms, could be interpreted in a more straightforward manner, and were ensured to be on a comparable scale [23]. The resultant coefficient estimates can be interpreted as the changes in Y associated with changes from low values to high values in the case of both binary and numeric predictors. It is important to note that because the dummy variables have been centered, they no longer have values of 0 and 1, but rather have positive and negative values between −1 and 1. Thus, in the presence of centered dummy variables, care must be given to the interpretation of interaction terms, since the input values are no longer 0 and 1.

We assessed our models using four criteria. First, for the SS algorithms, we determined if covariates were selected at different spatial scales, and tallied the number of covariates that were selected at each spatial scale. Second, we made AIC comparisons across three different settings: (1) when constraining all selected neighborhood-level covariates to enter a model at the CBG level; (2) when constraining all selected neighborhood-level covariates to enter a model at the CT level; and (3) when allowing all selected neighborhood-level covariates to enter a model at the original model-selected spatial scale (as determined by each SS algorithm). Third, to assess evaluating a random effect at different spatial scales, we compared AIC measures among the baseline SS main effects models (models fit with no RE) and the corresponding mixed models (models fit with REs). Fourth, we compared AIC values among the interaction models and the corresponding mixed models with interaction terms.

## 3. Results

### 3.1. Spatial Scale Main Effects Models

**Figure 2:**
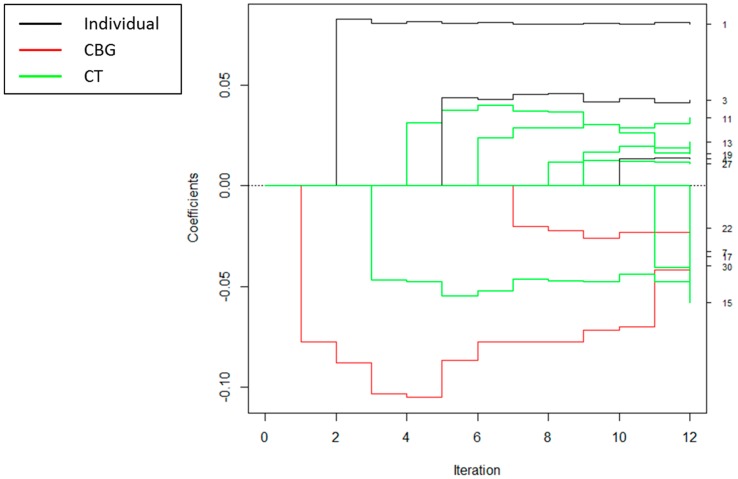
Coefficient paths for spatial scale forward stepwise regression to explain BMI z-scores. The scale at which each covariate entered the model is indicated by the legend. The numbers on the right-hand side of the figure are the variable numbers listed in Table 1.

**Figure 3:**
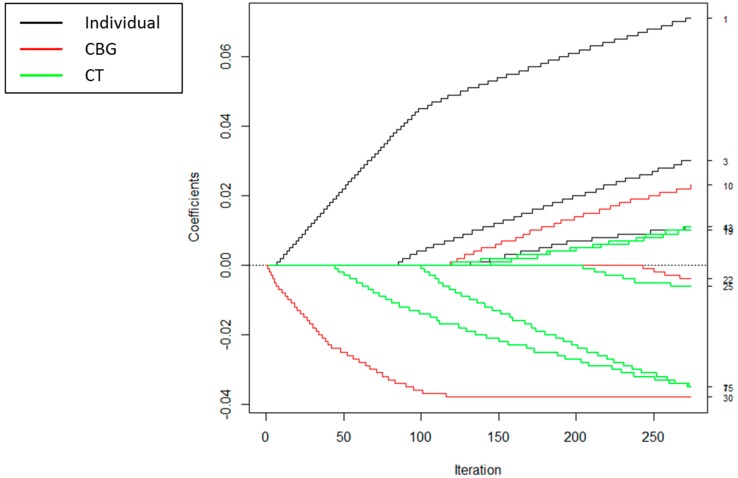
Coefficient paths for spatial scale incremental forward stagewise regression to explain BMI z-scores. The scale at which each covariate entered the model is indicated by the legend. The numbers on the right-hand side of the figure are the variable numbers listed in Table 1.

**Figure 4:**
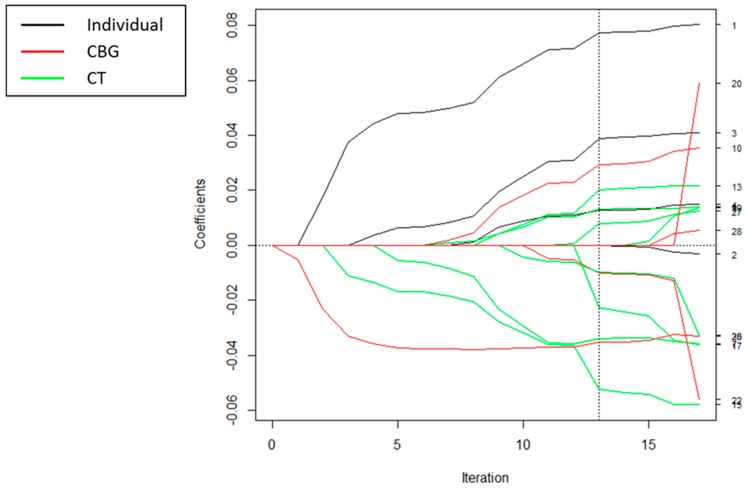
Coefficient paths for spatial scale least angle regression (LARS)/lasso to explain BMI z-scores. The scale at which each covariate entered the model is indicated by the legend. The numbers on the right-hand side of the figure are the variable numbers listed in Table 1. The dotted vertical line indicates the chosen model that had the minimum ordinary least squares (OLS)-based Akaike’s information criterion (AIC).

For each of the SS algorithms, covariates were selected at different spatial scales, as demonstrated in the coefficient path plots in Figure 2, Figure 3 and Figure 4. Each figure depicts the coefficient paths across the iterations of each algorithm. Black lines denote individual-level variables, red lines designate neighborhood variables selected at the census block group level, and green lines correspond to neighborhood variables selected at the census tract level. For the spatial scale forward stepwise model, the first covariate added was total expenditures per capita spent on sports/rec/exercise equipment at the CBG level, and the second variable added was individual visit age. For all of the algorithms, we chose to enter more variables at the larger CT level than at the smaller CBG level (Table 2). For these analyses, the SS LARS and SS lasso algorithms yielded the same set of solution paths; thus, we will refer to them jointly as SS LARS/lasso for the rest of this paper.

Table 3 reports the standardized coefficient estimates for covariates that were selected by each of the SS algorithms. Across all three SS algorithms, there was a significant positive relationship between BMI z-score and each of the following covariates (variable numbers are listed in parentheses): visit age (1), black (3), and percent Hispanic black at the CT level (13). The distance to the medical center was positively associated with BMIZ, and marginally significant for two of the three algorithms. There was a significant negative relationship between BMI z-score and the following covariates: population density at the CT level (7), median household income at the CT level (15), and total expenditures per capita spent on sports/recreation/exercise equipment at the CBG level (30).

The AIC comparisons for the three aforementioned model settings are given in Table 4. Model 3, where the spatial scale selection of area-level covariates was determined by each of the SS algorithms, had a substantially lower AIC than the other models where the spatial scale was fixed. We followed the rule of thumb of Burnham and Anderson [18] of an AIC difference of four to seven as indicating a meaningful difference in the fit of two models. There is no support for the model with the larger AIC when the difference between models exceeds 10 [18]. Thus, allowing covariates to enter a model at different spatial scales resulted in a significant improvement in the goodnes-of-fit. In addition, the SS stepwise and SS LARS/lasso models had better goodness-of-fit than the SS stagewise model.

OLS regression coefficient estimates are shown in Table 5, Table 6 and Table 7 for the models represented in Table 4. Each table compares the covariate effects for models with all area-level covariates at the (1) CBG level; (2) CT level; (3) and level selected by the SS algorithm. For each table, the sign of the effect estimates was the same across all three models. While the effect estimates related to BMI z-score were generally small, the relatively larger differences in the magnitude of effects were found between the CBG and CT models and the CBG and SS models. The estimates from the CT and SS models were more similar, because more variables were selected at the CT level than at the CBG level in the SS models (Table 2). Among the differences, there was a (0.04–0.06)/0.04 = 47% change in the median household income effect when going from the CBG scale assumption to the CT scale assumption with the forward stepwise algorithm (Table 5). The change was 35% in this effect, going from the CBG scale assumption to the SS model. The effect estimates are also plotted in the supplemental material (Figure S1). For the forward stagewise algorithm, the change in this effect was 64% from the CBG model to the CT model and was 57% from the CBG model to the SS model (Table 6). The percent changes in this effect are similar for the LARS/lasso models (Table 7). In this analysis, assuming the CBG scale leads to a reduced estimate of the association for median household income and childhood obesity. Another variable with a relatively large change in effect was exercise equipment expenditures, with a 38% change in the estimate from the CBG model to the CT model for the LARS/lasso algorithm. Given that one does not typically know which spatial scale to use a priori, the observed differences in effect estimates between the models provided some evidence of the utility of the SS algorithms and explained the differences in AICs between models.

### 3.2. Random Effects

A comparison of the SS algorithm estimates and the estimates obtained from mixed-effects models where REs were included at different spatial scales are shown in Table 8, Table 9 and Table 10. Inclusion of REs did not substantially alter the sign, magnitude, or significance of the coefficient estimates of the neighborhood-level covariates. Maps of the random effects for the Richmond MSA are included in the supplemental material (Figure S2) for each of the SS-based models. Maps featuring a RE at the CT highlight more tracts along the outskirts of the MSA as having higher BMI values. For example, the darkest census tract near the southernmost part of the MSA is a part of Dinwiddie County, a largely rural county. The increase in BMI may be reflective of the propensity of children and adolescents from rural areas to be more overweight [24,25].

The AIC measures (Table 11) of the SS stepwise, SS stagewise, and SS LARS/lasso models (fit with no RE) in comparison to their counterpart mixed models show that the SS models yielded higher AIC values than their corresponding random effect models, of which the mixed model with a RE at the CBG had the lowest AIC value. Thus, fitting a model with a random effect produced a better fit for BMI. Furthermore, adding a RE at the smaller CBG level produced the best results, indicating that people who live in the same CBG are more similar than those who live in the same CT in terms of BMI.

### 3.3. Interactions

Of the nine interaction terms that were included, the following four terms were statistically significant: Male × MHI_CT, Black × Population Density_CT, Black × Park Density_CT, and Black × Exercise Equipment_CBG. In order to obtain final models, we refit our models using only the significant interaction terms. The results for the final interaction models are presented in Table 12. Black children had a more negative slope for population density than white children. In other words, the negative association between population density and BMI z-score was enhanced for black children compared with white children. Black children had a less negative slope for exercise equipment than white children. In other words, the negative association between exercise equipment and BMI z-score was diminished for black children compared with white children. The negative association between median household income and BMI z-score was reduced for boys compared to girls. The positive association between park density and BMI z-score was greater for black children compared with white children.

As was done for the baseline SS models, we fit the interaction models with REs. A comparison of the coefficient estimates for the fixed effects showed that accounting for REs did not drastically change the signs, magnitude, or significance of the fixed-effect terms (Supplemental Tables S2–S4). As reflected in Table 13, an inspection of AIC values among interaction models fit with varying REs revealed that the models fit with a RE had smaller AIC values than models fit without a RE. Models with a RE at the smaller CBG level had the best goodness of fit with the lowest AIC measures; however, the differences in AIC among the RE models were small. The AIC values were substantially lower than those in Table 11, reflecting the addition of the significant interaction terms. Maps of the random effects of the interaction models had patterns similar to those observed for the random effects of the main effects models (Figure S3).

## 4. Discussion

In summary, we estimated the association between BMI z-score and both individual-level covariates and neighborhood-level covariates available at more than one spatial scale using our spatial scale selection algorithms. We found evidence of significant positive associations with visit age and black race at the individual level, percent Hispanic white at the census block group level, percent Hispanic black at the census tract level, and percent vacant housing at the census tract level. Conversely, we found significant negative associations with population density at the census tract level, median household income at the census tract level, percent renter at the census tract level, and exercise equipment expenditures at the census block group level. The negative association between population density and BMI z-score was stronger for black children compared with white children. The results of a positive association between percent vacant housing and BMI, and an inverse association of median household income and BMI, support earlier findings of a positive association between poverty at the ZIP code level and BMI [26], and community level disadvantage and BMI [27,28]. SES and weight generally have an inverse association at the individual level [29], and individuals with a higher SES tend to live in more affluent neighborhoods [26]. The inverse association between exercise equipment expenditures and BMI supports the idea that those living in more affluent areas have a greater ability to convert personal income into healthy physical activity [26].

Our modeling findings were two-fold: (1) modeling different neighborhood-level covariates at different spatial scales resulted in the best goodness-of-fit; and (2) adding a random effect at the smaller CBG level further improved model fit. The first finding aligns with what we previously observed when modeling variation in nitrate in private water wells [20]. The second finding is not surprising when one considers that individuals living in the same neighborhood may be similar in ways that our covariates did not measure, and that smaller spatial units are likely to be more homogeneous than larger spatial units [30].

A strength of this study was using methods that did not limit the evaluation of neighborhood factors to a single geographic level. Typically, past approaches have focused either on the most granular level available or the suggested level of geography based on the literature, e.g., census tract for SES variables, and then that is used for all variables. For applicability to inform public health programs and policy, our approach allows policy makers to understand at which level place-based factors are operating. For example, factors such as neighborhood safety and food access may be shaped by experiences within the block or block group, but access to other factors such as jobs or employment may be more relevant at the tract level. An important part of the precision public health movement is to empower policy makers to develop targeted policies and programs at the community level to improve health. An example would be to identify that the census block group is the best scale for a public health education campaign to be most effective.

While our analyses proved beneficial in establishing key findings, they were not without limitations. First, the covariates that were considered as candidates for model inclusion were limited in scope. For instance, only four individual-level variables were available; however, there are arguably many more covariates, such as nutrition and physical activity, which would be useful to include at the individual level. Furthermore, the neighborhood-level variable park density was calculated based on point locations, regardless of the park area, and only included parks that had the word “park” in the name. Therefore, our models were not exhaustive in explaining variation in BMI z-scores. In addition, there was potential for spurious variation in BMI due to measurement errors in height or weight. 

Opportunities for future work include the modification of our SS algorithms to consider interaction terms within each algorithm. Along with individual and area-level variables, interaction terms could be input as candidate variables for model inclusion. However, consideration would need to be given to events following the selection of an interaction term in order to ensure that the corresponding main effects terms are included as well if they have not already been selected. Thus, Efron et al. might perhaps give a better solution [31], as their work discusses checking for interactions after selecting the main effects by successively using the LARS algorithm: once to select the main effects terms, and again to select the interaction terms. For the second run, these authors recommended adjusting the outcome Y by subtracting the fitted values obtained from the main effects model, and using interaction terms as input variables for the design matrix X [31]. To incorporate the interactions within each SS algorithm, one could modify each SS algorithm to consider interactions after the main effects are selected by adopting the strategy presented by Efron et al. [31].

An additional idea for future work is the modification of our SS algorithms to adjust for confounders by forcing specific variables to be in a model. For example, the R package glmnet permits users to identify variables that they desire to always be in a model [32]. This is accomplished by ensuring that the coefficient estimates of the specified confounders are never shrunk to zero [33]. As a starting measure to address the issue of confounders within each SS algorithm, one could (1) initially set the coefficient estimates of the confounders equal to the corresponding OLS estimates; (2) subtract the fitted values from the outcome variable Y; and (3) proceed as usual through the remaining steps of each algorithm.

A final consideration for future work is the modification of our SS algorithms to implement random effect selection. After an algorithm selects the best scale at which to model each covariate, it could then select the best random effect based upon a minimum AIC criterion. Thus far, we have only examined independent random effects, but it would be worthwhile to explore spatially correlated random effects. The use of random effect selection may or may not be appropriate, depending on the data. However, in cases of data that is hierarchical in nature, random effect selection would be a useful tool in order to enhance model fit.

## 5. Conclusions

In this study, in order to explain variation in pediatric body mass index, we observed different socioeconomic covariates entered at different spatial scales in regression models using our spatial scale algorithms. This led to a better model fit in comparison to traditional models, where all area-level covariates were modeled at the same scale. In summary, these findings emphasized the importance of considering spatial scale when performing model selection. Based on our findings, we recommend the use of our SS algorithms over the generally accepted traditional modeling approaches of assuming a common spatial unit for all area-level covariates. The algorithms provide tools for investigators who wish to study contextual effects in public health research. 

## Figures and Tables

**Table 1:** Variables available for spatial scale selection algorithms. The horizontal dashed line separates the individual-level variables and the neighborhood-level variables available at more than one spatial scale. CBG: census block group, CBK: census block, CT: census tract, VCU: Virginia Commonwealth University.

Variable Number	Name	Description
1	VisitAge	Age at visit in years
2	Male	Indicator variable for male
3	Black	Indicator variable for black
4	MCVdist	Distance to VCU Medical Center (miles)
5–7	POPDENS	Population density (people/square mile) in 2010 CBK/CBG/CT
8–9	PBLACK	Percent of population that is black in CBG/CT
10–11	PHWHITE	Percent of population that is Hispanic white in CBG/CT
12–13	PHBLACK	Percent of population that is Hispanic black in CBG/CT
14–15	MEDHINC	Median household income in CBG/CT
16–17	PRENTER	Percent of households that are rented in CBG/CT
18–19	PVACANT	Percent of households that are vacant in CBG/CT
20–21	CRMCYTOTC	Total crime index in CBG/CT
22–23	CRMCYPERC	Crimes against persons index in CBG/CT
24–25	CRMCYPROC	Property crime index in CBG/CT
26–27	PARKDENS	Park density in CBG/CT
28–29	RESTDENS	Restaurant density in CBG/CT
30–31	EX_EQ	Expenditures per capita spent on sports/exercise equipment in CBG/CT

**Table 2:** Number of covariates selected at the individual-level and at each spatial scale (SS) by SS forward stepwise, forward stagewise, and LARS/lasso models. The last row lists the total number of possible variables at each data scale.

	Individual-Level	Area-Level			Number Selected
		CBK	CBG	CT	
SS Stepwise	3	0	2	7	12
SS Stagewise	3	0	3	5	11
SS LARS/Lasso	3	0	3	7	13
No. of available variables	4	13			17

**Table 3:** Standardized coefficient estimates from spatial scale (SS) forward stepwise, forward stagewise, LARS, and lasso models of BMIZ. The blank cells indicate variables not selected for a particular model. The horizontal dashed line separates the individual-level variables and the neighborhood-level variables considered at multiple spatial scales. The suffix CT or CBG indicates the spatial scale selected for the variable.

Explanatory Variable	SS Stepwise	SS Stagewise	SS LARS/Lasso
Visit Age	0.080 (*)	0.071 (*)	0.077 (*)
Black	0.043 (*)	0.030 (*)	0.039 (*)
Distance to Medical Center	0.013 (+)	0.011 (*)	0.013 (+)
Population Density_CT	−0.033 (*)	−0.035 (*)	−0.034 (*)
% Hispanic White_CBG		0.023 (*)	0.029 (*)
% Hispanic White_CT	0.034 (*)		
% Hispanic Black_CT	0.022 (*)	0.011 (*)	0.020 (*)
Median Household Income_CT	−0.058 (*)	−0.035 (*)	−0.052 (*)
% Renter_CT	−0.035 (*)		−0.023 (*)
% Vacant_CT	0.016 (*)	0.010 (*)	0.013 (+)
Personal Crime Index_CBG	−0.021 (*)	−0.004	−0.010
Property Crime Index_CT		−0.006	−0.010
Park Density_CT	0.011 (+)		0.008 (+)
Exercise Equipment_CBG	−0.040 (*)	−0.038 (*)	−0.035 (*)

Notes: Values marked with (*) have a *p*-value < 0.05, and values marked with (+) have an associated *p*-value < 0.1 (when covariates selected by the SS stagewise and SS LARS/lasso algorithms were plugged into OLS regression models).

**Table 4:** OLS-based Akaike’s information criterion comparisons across spatial scale (SS) forward stepwise, forward stagewise, and LARS/lasso models.

	SS Stepwise	SS Stagewise	SS LARS/Lasso
Model 1: CBG	77,666	77,673	77,668
Model 2: CT	77,658	77,668	77,657
Model 3: CBG and CT	77,648	77,657	77,647

**Table 5:** OLS coefficient estimates for three models based on covariates that were selected by the spatial scale (SS) forward stepwise model of BMI z-score: (1) when constraining all selected area-level covariates to enter at the CBG level; (2) when constraining all selected area-level covariates to enter at the CT level; and (3) when allowing all selected area-level covariates to enter at the model-selected spatial scale. The horizontal dashed line separates the individual-level variables and the area-level variables.

Explanatory Variable	CBG	CT	SS
Visit Age	0.080	0.080	0.080
Black	0.045	0.044	0.043
Distance to Medical Center	0.017	0.014	0.013
Population Density	−0.033	−0.033	−0.033 ^t^
% Hispanic White	0.035	0.035	0.034 ^t^
% Hispanic Black	0.020	0.021	0.022 ^t^
Median Household Income	−0.043	−0.063	−0.058 ^t^
% Renter	−0.028	−0.035	−0.035 ^t^
% Vacant	0.017	0.015	0.016 ^t^
Personal Crime Index	−0.020	−0.017	−0.021 ^b^
Park Density	0.007	0.011	0.011 ^t^
Exercise Equipment	−0.047	−0.032	−0.040 ^b^

Notes: Values marked with ^b^ denote area-level covariates selected at the CBG level, and values marked with ^t^ denote area-level covariates selected at the CT level.

**Table 6:** OLS coefficient estimates for three models based on covariates that were selected by the spatial scale (SS) forward stagewise model of BMI z-score: (1) when constraining all selected area-level covariates to enter at the CBG level; (2) when constraining all selected area-level covariates to enter at the CT level; and (3) when allowing all selected area-level covariates to enter at the model-selected spatial scale. The horizontal dashed line separates the individual-level variables and the area-level variables.

Variable	CBG	CT	SS
Visit Age	0.081	0.081	0.081
Black	0.044	0.042	0.042
Distance to Medical Center	0.017	0.014	0.014
Population Density	−0.043	−0.048	−0.047 ^t^
% Hispanic White	0.035	0.032	0.031 ^b^
% Hispanic Black	0.014	0.016	0.019 ^t^
Median Household Income	−0.028	−0.046	−0.044 ^t^
% Vacant	0.018	0.017	0.017 ^t^
Personal Crime Index	−0.019	−0.010	−0.014 ^b^
Property Crime Index	−0.001	−0.011	−0.009 ^t^
Exercise Equipment	−0.050	−0.032	−0.036 ^b^

Notes: Values marked with ^b^ denote area-level covariates selected at the CBG level, and values marked with ^t^ denote area-level covariates selected at the CT level.

**Table 7:** OLS coefficient estimates for three models based on covariates that were selected by the spatial scale (SS) LARS/lasso model of BMI z-score: (1) when constraining all selected area-level covariates to enter at the CBG level; (2) when constraining all selected area-level covariates to enter at the CT level; and (3) when allowing all selected area-level covariates to enter at the model-selected spatial scale. The horizontal dashed line separates the individual-level variables and the area-level variables.

Explanatory Variable	CBG	CT	SS
Visit Age	0.080	0.080	0.081
Black	0.045	0.044	0.043
Distance to Medical Center	0.017	0.014	0.014
Population Density	−0.033	−0.033	−0.033 ^t^
% Hispanic White	0.035	0.034	0.033 ^b^
% Hispanic Black	0.020	0.022	0.025 ^t^
Median Household Income	−0.044	−0.065	−0.061 ^t^
% Renter	−0.028	−0.037	−0.035 ^t^
% Vacant	0.017	0.014	0.015 ^t^
Personal Crime Index	−0.018	−0.007	−0.013 ^b^
Property Crime Index	−0.002	−0.014	−0.012 ^t^
Park Density	0.007	0.011	0.012 ^t^
Exercise Equipment	−0.047	−0.029	−0.034 ^b^

Notes: Values marked with ^b^ denote area-level covariates selected at the CBG level, and values marked with ^t^ denote area-level covariates selected at the CT level.

**Table 8:** Standardized coefficient estimates from the spatial scale (SS) forward stepwise model of BMI z-scores with no random effect (RE) in comparison to standardized coefficient estimates from three mixed models with a RE at the census block group (CBG), the census tract (CT), and the CBG and CT. The mixed models were fit using the covariates that were chosen by the SS stepwise algorithm. The horizontal dashed line separates the individual-level variables and the neighborhood-level variables considered at multiple spatial scales.

Explanatory Variable	No RE	RE at CBG	RE at CT	RE at CBG and CT
Visit Age	0.080 (*)	0.081 (*)	0.081 (*)	0.081 (*)
Black	0.043 (*)	0.042 (*)	0.042 (*)	0.042 (*)
Distance to Medical Center	0.013 (+)	0.014 (+)	0.013 (+)	0.014 (+)
Population Density_CT	−0.033 (*)	−0.028 (*)	−0.028 (*)	−0.027 (*)
% Hispanic White_CT	0.034 (*)	0.032 (*)	0.032 (*)	0.032 (*)
% Hispanic Black_CT	0.022 (*)	0.016 (+)	0.019 (*)	0.017 (+)
Median Household Income_CT	−0.058 (*)	−0.052 (*)	−0.053 (*)	−0.052 (*)
% Renter_CT	−0.035 (*)	−0.031 (*)	−0.030 (*)	−0.030 (*)
% Vacant_CT	0.016 (*)	0.016 (+)	0.018 (*)	0.017 (*)
Personal Crime Index_CBG	−0.021 (*)	−0.021 (*)	−0.024 (*)	−0.022 (*)
Park Density_CT	0.011 (+)	0.010	0.010	0.009
Exercise Equipment_CBG	−0.040 (*)	−0.046 (*)	−0.043 (*)	−0.046 (*)

Notes: Values marked with (*) have a *p*-value < 0.05, and values marked with (+) have an associated *p*-value < 0.1.

**Table 9:** Standardized coefficient estimates from the spatial scale (SS) forward stagewise model of BMI z-scores with no random effect (RE) in comparison to standardized coefficient estimates from three mixed models with a RE at the census block group (CBG), the census tract (CT), and the CBG and CT. The mixed models were fit using the covariates that were chosen by the SS stagewise algorithm. The horizontal dashed line separates the individual-level variables and the neighborhood-level variables considered at multiple spatial scales.

Explanatory Variable	No RE	RE at CBG	RE at CT	RE at CBG and CT
Visit Age	0.071 (*)	0.081 (*)	0.081 (*)	0.081 (*)
Black	0.030 (*)	0.041 (*)	0.041 (*)	0.041 (*)
Distance to Medical Center	0.011 (*)	0.015 (+)	0.014 (+)	0.014 (+)
Population Density_CT	−0.035 (*)	−0.039 (*)	−0.038 (*)	−0.037 (*)
% Hispanic White_CBG	0.023 (*)	0.029 (*)	0.030 (*)	0.029 (*)
% Hispanic Black_CT	0.011 (*)	0.013	0.016 (+)	0.013
Median Household Income_CT	−0.035 (*)	−0.040 (*)	−0.042 (*)	−0.040 (*)
% Vacant_CT	0.010 (*)	0.017 (*)	0.019 (*)	0.018 (*)
Personal Crime Index_CBG	−0.004	−0.015	−0.018	−0.016
Property Crime Index_CT	−0.006	−0.010	−0.009	−0.009
Exercise Equipment_CBG	−0.038 (*)	−0.044 (*)	−0.040 (*)	−0.044 (*)

Notes: Values marked with (*) have a *p*-value < 0.05, and values marked with (+) have an associated *p*-value < 0.1. For the SS stagewise model with no RE, covariates selected by the SS stagewise algorithm were plugged into an OLS regression model to obtain estimated *p*-values.

**Table 10:** Standardized coefficient estimates from the spatial scale (SS) LARS/lasso model of BMI z-scores with no random effect (RE) in comparison to standardized coefficient estimates from three mixed models with a RE at the census block group (CBG), the census tract (CT), and the CBG and CT. The mixed models were fit using the covariates that were chosen by the SS LARS/lasso algorithm. The horizontal dashed line separates the individual-level variables and the neighborhood-level variables considered at multiple spatial scales.

Explanatory Variable	No RE	RE at CBG	RE at CT	RE at CBG and CT
Visit Age	0.077 (*)	0.081 (*)	0.081 (*)	0.081 (*)
Black	0.039 (*)	0.042 (*)	0.042 (*)	0.042 (*)
Distance to Medical Center	0.013 (+)	0.014 (+)	0.014 (+)	0.014 (+)
Population Density_CT	−0.034 (*)	−0.028 (*)	−0.028 (*)	−0.027 (*)
% Hispanic White_CBG	0.029 (*)	0.031 (*)	0.031 (*)	0.031 (*)
% Hispanic Black_CT	0.020 (*)	0.020 (*)	0.022 (*)	0.020 (*)
Median Household Income_CT	−0.052 (*)	−0.055 (*)	−0.056 (*)	−0.055 (*)
% Renter_CT	−0.023 (*)	−0.031 (*)	−0.030 (*)	−0.030 (*)
% Vacant_CT	0.013 (+)	0.015 (+)	0.016 (*)	0.015 (+)
Personal Crime Index_CBG	−0.010	−0.013	−0.016	−0.014
Property Crime Index_CT	−0.010	−0.012	−0.011	−0.012
Park Density_CT	0.008 (+)	0.010	0.010	0.010
Exercise Equipment_CBG	−0.035 (*)	−0.041 (*)	−0.038 (*)	−0.041 (*)

Notes: Values marked with (*) have a *p*-value < 0.05, and values marked with (+) have an associated *p*-value < 0.1. For the SS LARS/lasso model with no RE, covariates selected by the SS LARS/lasso algorithm were plugged into an OLS regression model to obtain estimated *p*-values.

**Table 11:** AIC comparisons among spatial scale (SS) forward stepwise, forward stagewise, and LARS/lasso models with varying random effects (RE): no RE, RE at CBG, RE at CT, and RE at CBG and CT.

	SS Stepwise	SS Stagewise	SS LARS/Lasso
No RE	77,648	77,657	77,647
RE at CBG	77,636	77,640	77,635
RE at CT	77,640	77,643	77,639
RE at CBG and CT	77,637	77,640	77,638

**Table 12:** Standardized coefficient estimates when the covariate male, select interaction terms, and covariates selected by the spatial scale (SS) forward stepwise, forward stagewise, and LARS/lasso algorithms were plugged into OLS regression models of BMI z-scores. The blank cells indicate variables not selected for a particular model. The horizontal dashed line separates the individual-level variables and the neighborhood-level variables considered at multiple spatial scales.

Explanatory Variable	SS Stepwise	SS Stagewise	SS LARS/Lasso
Intercept	0.584 (*)	0.587 (*)	0.584 (*)
Visit Age	0.161 (*)	0.163 (*)	0.161 (*)
Male	−0.006	−0.006	−0.006
Black	0.084 (*)	0.085 (*)	0.085 (*)
Distance to Medical Center	0.023	0.023	0.023
Population Density_CT	−0.038 (*)	−0.052 (*)	−0.038 (*)
% Hispanic White_CBG		0.038 (*)	0.041 (*)
% Hispanic White_CT	0.040 (*)		
% Hispanic Black_CT	0.039 (*)	0.035 (*)	0.042 (*)
Median Household Income_CT	−0.070 (*)	−0.053 (+)	−0.075 (*)
% Renter_CT	−0.034		−0.035
% Vacant_CT	0.043 (*)	0.043 (*)	0.041 (*)
Personal Crime Index_CBG	−0.039 (*)	−0.017	−0.024
Property Crime Index_CT		−0.021	−0.021
Park Density_CT	0.010		0.011
Exercise Equipment_CBG	−0.078 (*)	−0.071 (*)	−0.071 (*)
Male:MHI_CT	0.097 (*)	0.097 (*)	0.097 (*)
Black:Population Density_CT	−0.073 (*)	−0.062 (*)	−0.073 (*)
Black:Park Density_CT	0.079 (*)		0.078 (*)
Black:Exercise Equipment_CBG	0.167 (*)	0.185 (*)	0.167 (*)

Notes: Values marked with (*) have a *p*-value < 0.05, and values marked with (+) have an associated *p*-value < 0.1.

**Table 13:** AIC comparisons among SS forward stepwise-, SS forward stagewise, and SS LARS/lasso-based models with interactions and varying random effects (RE): no RE, RE at CBG, RE at CT, and RE at CBG and CT.

	SS Stepwise	SS Stagewise	SS LARS/Lasso
No RE	77,128	77,137	77,127
RE at CBG	77,123	77,131	77,123
RE at CT	77,126	77,133	77,125
RE at CBG and CT	77,125	77,132	77,124

## Supplementary Materials

The following are available online at http://www.mdpi.com/1660-4601/15/3/473/s1, Figure S1: individual and area-level effect estimates from models with the area-level covariates all at the census block group (CBG) level, census tract (CT) level, or spatial scale (SS) level selected by the SS forward stepwise algorithm, Figure S2: maps of random effects at the census block group (CBG), census tract (CT), and CBG/CT for linear mixed-effects models of BMIZ using covariates selected by the spatial scale (SS) forward stepwise, forward stagewise, and LARS/lasso algorithms in the Richmond metropolitan statistical area, Figure S3: maps of random effects at the census block group (CBG), census tract (CT), and CBG/CT for linear mixed-effects models of BMIZ using covariates selected by the spatial scale (SS) forward stepwise, forward stagewise, and LARS/lasso algorithms, and including the covariate male and select interaction terms in the Richmond metropolitan statistical area (MSA), Table S1: reference table for interpretation of the slopes under four different conditions, where different signs are realized by coefficient estimates for a continuous main effect $\left({\hat{\beta }}_{2}\right)$ and an interaction $\left({\hat{\beta }}_{12}\right)$, Table S2: standardized coefficient estimates when the covariate male, select interaction terms, and covariates selected by the spatial scale forward stepwise algorithm were plugged into an OLS regression model of BMIZ with no random effect (RE), in contrast to coefficient estimates from three mixed models with a RE at the census block group (CBG), RE at the census tract (CT), and RE at the CBG and CT. The horizontal dashed line separates the individual-level variables, and the neighborhood-level variables were considered at multiple spatial scales, Table S3: standardized coefficient estimates when the covariate male, select interaction terms, and covariates selected by the spatial scale forward stagewise algorithm were plugged into an OLS regression model of BMIZ with no random effect (RE) in contrast to coefficient estimates from three mixed models with a RE at the census block group (CBG), RE at the census tract (CT), and RE at the CBG and CT. The horizontal dashed line separates the individual-level variables and the neighborhood-level variables considered at multiple spatial scales, Table S4: standardized coefficient estimates when the covariate male, select interaction terms, and covariates selected by the spatial scale LARS/lasso algorithm were plugged into an OLS regression model of BMIZ with no random effect (RE) in contrast to coefficient estimates from three mixed models with a RE at the census block group (CBG), RE at the census tract (CT), and RE at the CBG and CT. The horizontal dashed line separates the individual-level variables and the neighborhood-level variables considered at multiple spatial scales.

## Appendix A

### Appendix A.1. R Code for Data Processing

#### Pediatric BMI dataset with merged attribute data ####

mydata <- mydatasp_and_attribute.CBG_and_CT_bw[,c("BMIZ", "Visit_Age", "Male", "Black", "MCV.dist", "cbk_POPDENS10", "cbg_POPDENS10", "ct_POPDENS10", "cbg_PBLACK10", "ct_PBLACK10", "cbg_PHWHITE10", "ct_PHWHITE10", "cbg_PHBLACK10", "ct_PHBLACK10", "cbg_MEDHINC_CY", "ct_MEDHINC_CY", "cbg_PRENTER10", "ct_PRENTER10", "cbg_PVACANT10", "ct_PVACANT10", "cbg_CRMCYTOTC", "ct_CRMCYTOTC", "cbg_CRMCYPERC", "ct_CRMCYPERC", "cbg_CRMCYPROC", "ct_CRMCYPROC",

"cbg_PARKDENS", "ct_PARKDENS", "cbg_RESTDENS", "ct_RESTDENS", "cbg_ex_eq", "ct_ex_eq")]

# Standardize (center and scale) variables

library(gtools)

zscore <- defmacro(data, var, expr={data$var <- (data$var-mean(data$var, na.rm = TRUE))/sd(data$var, na.rm = TRUE)})

zscore(mydata, BMIZ); zscore(mydata, Visit_Age); zscore(mydata, Male); zscore(mydata, Black); zscore(mydata, MCV.dist);

zscore(mydata, cbk_POPDENS10); zscore(mydata, cbg_POPDENS10); zscore(mydata, ct_POPDENS10);

zscore(mydata, cbg_PBLACK10); zscore(mydata, ct_PBLACK10);

zscore(mydata, cbg_PHWHITE10); zscore(mydata, ct_PHWHITE10);

zscore(mydata, cbg_PHBLACK10); zscore(mydata, ct_PHBLACK10);

zscore(mydata, cbg_MEDHINC_CY); zscore(mydata, ct_MEDHINC_CY);

zscore(mydata, cbg_PRENTER10); zscore(mydata, ct_PRENTER10);

zscore(mydata, cbg_PVACANT10); zscore(mydata, ct_PVACANT10);

zscore(mydata, cbg_CRMCYTOTC); zscore(mydata, ct_CRMCYTOTC);

zscore(mydata, cbg_CRMCYPERC); zscore(mydata, ct_CRMCYPERC);

zscore(mydata, cbg_CRMCYPROC); zscore(mydata, ct_CRMCYPROC);

zscore(mydata, cbg_PARKDENS); zscore(mydata, ct_PARKDENS);

zscore(mydata, cbg_RESTDENS); zscore(mydata, ct_RESTDENS);

zscore(mydata, cbg_ex_eq); zscore(mydata, ct_ex_eq);

y <- mydata[,1]

X <- mydata[,-1]

names.X <- colnames(X)

y.name <- "BMIZ"

list.names <- c("Visit_Age", "Male", "Black", "MCV.dist", "POPDENS10", "PBLACK10", "PHWHITE10", "PHBLACK10", "MEDHINC_CY", "PRENTER10", "PVACANT10",

"CRMCYTOTC", "CRMCYPERC", "CRMCYPROC", "PARKDENS", "RESTDENS", "ex_eq")

NP <- dim(X)

N <- NP[1]

P <- length(list.names)

K <- 4

X.3D <- array(NA, dim=c(N,P,K), dimnames=list(NULL, list.names, NULL))

X.3D[,1,1] <- X[, 1]

X.3D[,2,1] <- X[, 2]

X.3D[,3,1] <- X[, 3]

X.3D[,4,1] <- X[, 4]

X.3D[,5,2] <- X[, 5]

X.3D[,5,3] <- X[, 6]

X.3D[,6,3] <- X[, 8]

X.3D[,7,3] <- X[, 10]

X.3D[,8,3] <- X[, 12]

X.3D[,9,3] <- X[, 14]

X.3D[,10,3] <- X[, 16]

X.3D[,11,3] <- X[, 18]

X.3D[,12,3] <- X[, 20]

X.3D[,13,3] <- X[, 22]

X.3D[,14,3] <- X[, 24]

X.3D[,15,3] <- X[, 26]

X.3D[,16,3] <- X[, 28]

X.3D[,17,3] <- X[, 30]

X.3D[,5,4] <- X[, 7]

X.3D[,6,4] <- X[, 9]

X.3D[,7,4] <- X[, 11]

X.3D[,8,4] <- X[, 13]

X.3D[,9,4] <- X[, 15]

X.3D[,10,4] <- X[, 17]

X.3D[,11,4] <- X[, 19]

X.3D[,12,4] <- X[, 21]

X.3D[,13,4] <- X[, 23]

X.3D[,14,4] <- X[, 25]

X.3D[,15,4] <- X[, 27]

X.3D[,16,4] <- X[, 29]

X.3D[,17,4] <- X[, 31]

ss <- c("none", "cbk", "cbg", "ct")

a.lst <- list(NULL)

a.lst[[1]] <- 1

dim(a.lst[[1]]) <- c(1,1)

dimnames(a.lst[[1]]) <- list(NULL, names.X[1])

a.lst[[2]] <- 1

dim(a.lst[[2]]) <- c(1,1)

dimnames(a.lst[[2]]) <- list(NULL, names.X[2])

a.lst[[3]] <- 1

dim(a.lst[[3]]) <- c(1,1)

dimnames(a.lst[[3]]) <- list(NULL, names.X[3])

a.lst[[4]] <- 1

dim(a.lst[[4]]) <- c(1,1)

dimnames(a.lst[[4]]) <- list(NULL, names.X[4])

a.lst[[5]] <- diag(3)

dimnames(a.lst[[5]]) <- list(NULL, names.X[c(5:7)])

for (j in 6:17) {

a.lst[[j]] <- diag(2)

start <- 2*j-4

dimnames(a.lst[[j]]) <- list(NULL, names.X[c(start, start+1)])

}

S.v <- NULL

for (j in 1:length(list.names)) {

if (length(a.lst[[j]])==1) {

S.v[j] <- length(a.lst[[j]])

} else if (length(a.lst[[j]])!=1) {

S.v[j] <- dim(a.lst[[j]])[2]

}

}

C.v <- rep(0,length(list.names))

col.plot <- c("black", "blue", "red", "green")

### Appendix A.2. R Code for Spatial Scale Algorithms

library(spselect)

### Spatial scale forward stepwise regression ###

mod_forward.step.ss_1 <- stepwise.ss(y, X.3D, y.name, ss, epsilon=1)

mod_forward.step.ss_1$beta.final *# 12 vars*

table(mod_forward.step.ss_1$stack.ss)

### Spatial scale forward stagewise regression ###

mod_forward.stage.ss_0.01 <- stagewise.ss(y, X, X.3D, ss, increment=0.001, tolerance=0.01, col.plot)

mod_forward.stage.ss_0.01$beta.final *# 11 vars*

table(mod_forward.stage.ss_0.01$stack.ss)

### Spatial scale LARS ###

mod_LARS.ss <- lars.ss(y, X, ss, a.lst, S.v, C.v, col.plot)

round(mod_LARS.ss$beta.aic, 3) *# 13 vars*

### Spatial scale lasso ###

mod_lasso.ss <- lasso.ss(y, X, ss, a.lst, S.v, C.v, col.plot)

round(mod_lasso.ss$beta.aic, 3) *# 13 vars*

table(round(mod_LARS.ss$beta.aic-mod_lasso.ss$beta.aic,6))
