# Supplementary material for: Modeling Pediatric Body Mass Index and Neighborhood Environment at Different Spatial Scales
Source: Int J Environ Res Public Health. 2018 Mar 8;15(3):473. doi: 10.3390/ijerph15030473 (PMC5877018; doi:10.3390/ijerph15030473)
Supplement: Supplementary file 1 [file ijerph-15-00473-s001.pdf]

Modeling pediatric BMI and neighborhood environment at different spatial scales

Supplementary Material

Lauren P. Grant, Chris Gennings, Edmond P. Wickham III, Derek Chapman, Shumei Sun, David C. Wheeler

Covariate Effects

The individual and area-level effect estimates from models with the area-level covariates all at the census block group (CBG) level, census tract (CT) level, or spatial scale (SS) level selected by the SS forward stepwise algorithm are plotted in Figure S1.

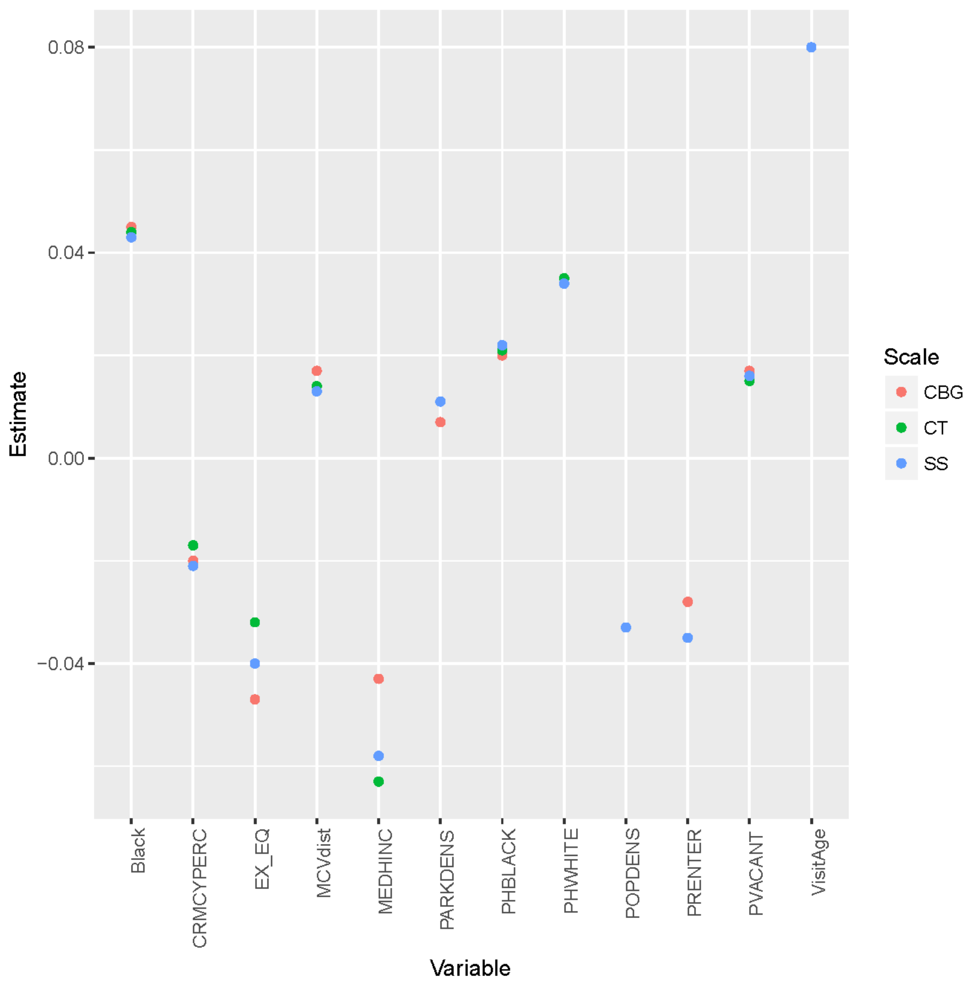

Figure S1. Individual and area-level effect estimates from models with the area-level covariates all at the census block group (CBG) level, census tract (CT) level, or spatial scale (SS) level selected by the SS forward stepwise algorithm.

Random Effects

Across the SS-based models, the maps (Figure S2) featuring a random effect (RE) at the CBG and REs at both the CBG and the CT appear similar, while the maps featuring a RE at the CT appear to highlight more tracts along the outskirts of the MSA as having higher BMI values.

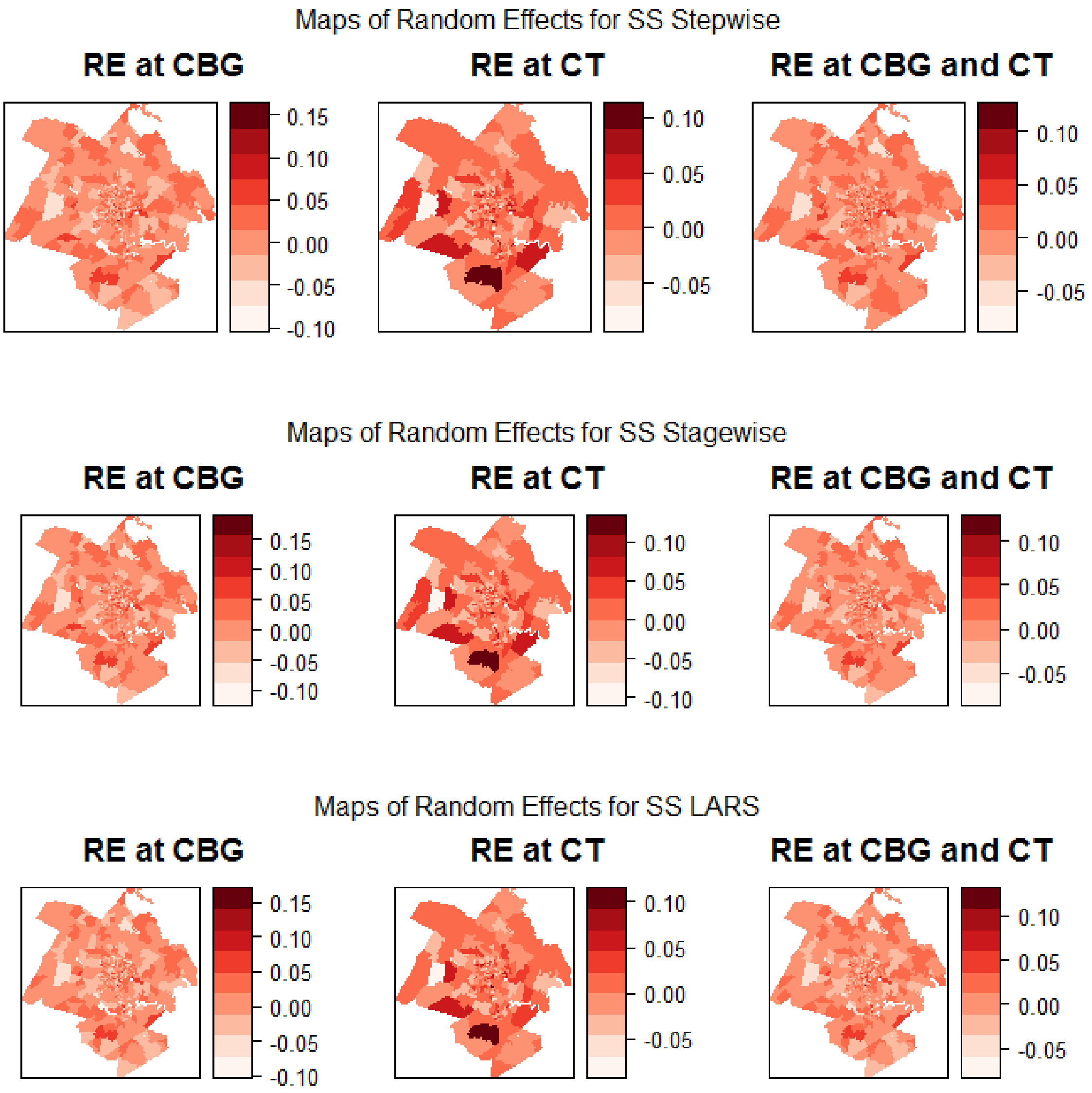

Figure S2. Maps of random effects at the census block group (CBG), census tract (CT), and CBG/CT for linear mixed-effects models of BMIZ using covariates selected by the spatial scale (SS) forward stepwise, forward stagewise, and LARS/lasso algorithms in the Richmond Metropolitan Statistical Area.

Interactions

For the sake of example, let $\boldsymbol{x}_d$ be a centered dummy variable, where $\boldsymbol{x}_d = \begin{cases} d_0 < 0 \\ d_1 > 0 \end{cases}$, and let $\boldsymbol{x}_c$ be a standardized continuous variable. If we are interested in including an interaction between a binary and continuous variable, then the regression equation can be written as follows:

$$\boldsymbol{y} = \beta_0 + \beta_1 \boldsymbol{x}_d + \beta_2 \boldsymbol{x}_c + \beta_{12} \boldsymbol{x_d} \boldsymbol{x_c} + \boldsymbol{\varepsilon}.$$

When the reference group $x_d = d_0$, the slope is:

$$\beta_2 + \beta_{12} d_0,$$

and when the non-reference group $x_d = d_1$, the slope is

$$\beta_2 + \beta_{12} d_1.$$

Depending on whether the estimates for β_2 and β_{12} are positive or negative will affect the interpretation of the two slopes relative to one another. To aid in the interpretation of the interaction effect, Table S1 provides general guidelines for interpretation of the slopes under four different conditions.

Table S1. Reference table for interpretation of the slopes under four different conditions, where different signs are realized by coefficient estimates for a continuous main effect ($\hat{\beta}_2$) and an interaction ($\hat{\beta}_{12}$).

Case	$\hat{\beta}_2$	$\hat{\beta}_{12}$	Interpretation of Interaction Effect
1	+	+	Non-reference group has more positive slope than reference group.
2	+	−	Non-reference group has less positive slope than reference group.
3	−	+	Non-reference group has less negative slope than reference group.
4	−	−	Non-reference group has more negative slope than reference group.

To illustrate the use of Table S1, let us consider two different examples: 1) the interaction between black and population density and 2) the interaction between black and exercise equipment as shown in Table 9. For the first example, observe that the main effect for population density had a negative sign, and the interaction between black and population density had a negative sign. Referring to Table S1, we can see that this falls under the realm of case 4. Thus, we can conclude that black children had a more negative slope for population density than white children. In other words, the negative association between population density and BMI *z*-score

was enhanced for black children as compared to white children. For the second example, note that the main effect for exercise equipment had a negative sign, while the interaction between black and exercise equipment had a positive sign. Thus, referring to Table S1, we can see that this falls under the realm of case 3. Thus, we can conclude that black children had a less negative slope for exercise equipment than white children. In other words, the negative association between exercise equipment and BMI *z*-score was diminished for black children as compared to white children.

Interactions and Random Effects

Regression coefficients for models with interaction terms and REs are listed in Tables S2-S4. Maps of the random effects for these models are in Figure S3.

Table S2. Standardized coefficient estimates when the covariate male, select interaction terms, and covariates selected by the spatial scale forward stepwise algorithm were plugged into an OLS regression model of BMIZ with no random effect (RE) in contrast to coefficient estimates from three mixed models with a RE at the census block group (CBG), RE at the census tract (CT), and RE at the CBG and CT. The horizontal dashed line separates the individual-level variables and the neighborhood-level variables considered at multiple spatial scales.

Explanatory Variable	No RE	RE at CBG	RE at CT	RE at CBG and CT
Intercept	0.584 (*)	0.582 (*)	0.583 (*)	0.582 (*)
Visit Age	0.161 (*)	0.161 (*)	0.161 (*)	0.161 (*)
Male	-0.006	-0.006	-0.006	-0.006
Black	0.084 (*)	0.082 (*)	0.081 (*)	0.081 (*)
Distance to Medical Center	0.023	0.024	0.023	0.024
Population Density_CT	-0.038 (*)	-0.037 (+)	-0.037 (+)	-0.036 (+)
% Hispanic White_CT	0.040 (*)	0.041 (*)	0.041 (*)	0.041 (*)
% Hispanic Black_CT	0.039 (*)	0.033 (+)	0.037 (*)	0.033 (+)
Median Household Income_CT	-0.070 (*)	-0.066 (*)	-0.068 (*)	-0.066 (*)
% Renter_CT	-0.034	-0.033	-0.030	-0.032
% Vacant_CT	0.043 (*)	0.042 (*)	0.045 (*)	0.043 (*)
Personal Crime Index_CBG	-0.039 (*)	-0.038 (*)	-0.043 (*)	-0.040 (*)
Park Density_CT	0.010	0.009	0.009	0.009

Exercise Equipment_CBG	-0.078 (*)	-0.087 (*)	-0.080 (*)	-0.085 (*)
Male:MHI_CT	0.097 (*)	0.097 (*)	0.097 (*)	0.097 (*)
Black:Population Density_CT	-0.073 (*)	-0.073 (*)	-0.072 (*)	-0.072 (*)
Black:Park Density_CT	0.079 (*)	0.079 (*)	0.080 (*)	0.079 (*)
Black:Exercise Equipment_CBG	0.167 (*)	0.155 (*)	0.160 (*)	0.154 (*)

Notes: Values marked with (*) have a *p*-value < 0.05, and values marked with (+) have an associated *p*-value < 0.1

Table S3. Standardized coefficient estimates when the covariate male, select interaction terms, and covariates selected by the spatial scale forward stagewise algorithm were plugged into an OLS regression model of BMIZ with no random effect (RE) in contrast to coefficient estimates from three mixed models with a RE at the census block group (CBG), RE at the census tract (CT), and RE at the CBG and CT. The horizontal dashed line separates the individual-level variables and the neighborhood-level variables considered at multiple spatial scales.

Explanatory Variable	No RE	RE at CBG	RE at CT	RE at CBG and CT
Intercept	0.587 (*)	0.585 (*)	0.585 (*)	0.584 (*)
Visit Age	0.163 (*)	0.163 (*)	0.163 (*)	0.163 (*)
Male	-0.006	-0.006	-0.006	-0.006
Black	0.085 (*)	0.083 (*)	0.083 (*)	0.083 (*)
Distance to Medical Center	0.023	0.024	0.023	0.023
Population Density_CT	-0.052 (*)	-0.050 (*)	-0.049 (*)	-0.049 (*)
% Hispanic White_CBG	0.038 (*)	0.039 (*)	0.040 (*)	0.039 (*)
% Hispanic Black_CT	0.035 (*)	0.029 (+)	0.033 (+)	0.029 (+)
Median Household Income_CT	-0.053 (+)	-0.051 (+)	-0.054 (+)	-0.052 (+)
% Vacant_CT	0.043 (*)	0.042 (*)	0.045 (*)	0.043 (*)
Personal Crime Index_CBG	-0.017	-0.017	-0.022	-0.019
Property Crime Index_CT	-0.021	-0.022	-0.020	-0.021
Exercise Equipment_CBG	-0.071 (*)	-0.081 (*)	-0.074 (*)	-0.079 (*)
Male:MHI_CT	0.097 (*)	0.097 (*)	0.098 (*)	0.097 (*)
Black:Population Density_CT	-0.062 (*)	-0.061 (*)	-0.060 (*)	-0.060 (*)
Black:Exercise Equipment_CBG	0.185 (*)	0.168 (*)	0.170 (*)	0.166 (*)

Notes: Values marked with (*) have a *p*-value < 0.05, and values marked with (+) have an associated *p*-value < 0.1

Table S4. Standardized coefficient estimates when the covariate male, select interaction terms, and covariates selected by the spatial scale LARS/lasso algorithm were plugged into an OLS regression model of BMIZ with no random effect (RE) in contrast to coefficient estimates from three mixed models with a RE at the census block group (CBG), RE at the census tract (CT), and RE at the CBG and CT. The horizontal dashed line separates the individual-level variables and the neighborhood-level variables considered at multiple spatial scales.

Explanatory Variable	No RE	RE at CBG	RE at CT	RE at CBG and CT
Intercept	0.584 (*)	0.582 (*)	0.583 (*)	0.582 (*)
Visit Age	0.161 (*)	0.161 (*)	0.161 (*)	0.162 (*)
Male	-0.006	-0.006	-0.005	-0.006
Black	0.085 (*)	0.083 (*)	0.083 (*)	0.082 (*)
Distance to Medical Center	0.023	0.023	0.022	0.023
Population Density_CT	-0.038 (*)	-0.036 (+)	-0.037 (+)	-0.036 (+)
% Hispanic White_CBG	0.041 (*)	0.041 (*)	0.042 (*)	0.042 (*)
% Hispanic Black_CT	0.042 (*)	0.036 (*)	0.040 (*)	0.037 (*)
Median Household Income_CT	-0.075 (*)	-0.071 (*)	-0.073 (*)	-0.071 (*)
% Renter_CT	-0.035	-0.035	-0.031	-0.033
% Vacant_CT	0.041 (*)	0.040 (*)	0.043 (*)	0.041 (*)
Personal Crime Index_CBG	-0.024	-0.022	-0.028	-0.024
Property Crime Index_CT	-0.021	-0.022	-0.020	-0.022
Park Density_CT	0.011	0.010	0.010	0.010
Exercise Equipment_CBG	-0.071 (*)	-0.079 (*)	-0.072 (*)	-0.078 (*)
Male:MHI_CT	0.097 (*)	0.097 (*)	0.097 (*)	0.097 (*)
Black:Population Density_CT	-0.073 (*)	-0.073 (*)	-0.072 (*)	-0.072 (*)
Black:Park Density_CT	0.078 (*)	0.078 (*)	0.079 (*)	0.079 (*)
Black:Exercise Equipment_CBG	0.167 (*)	0.154 (*)	0.159 (*)	0.154 (*)

Notes: Values marked with (*) have a *p*-value < 0.05, and values marked with (+) have an associated *p*-value < 0.1

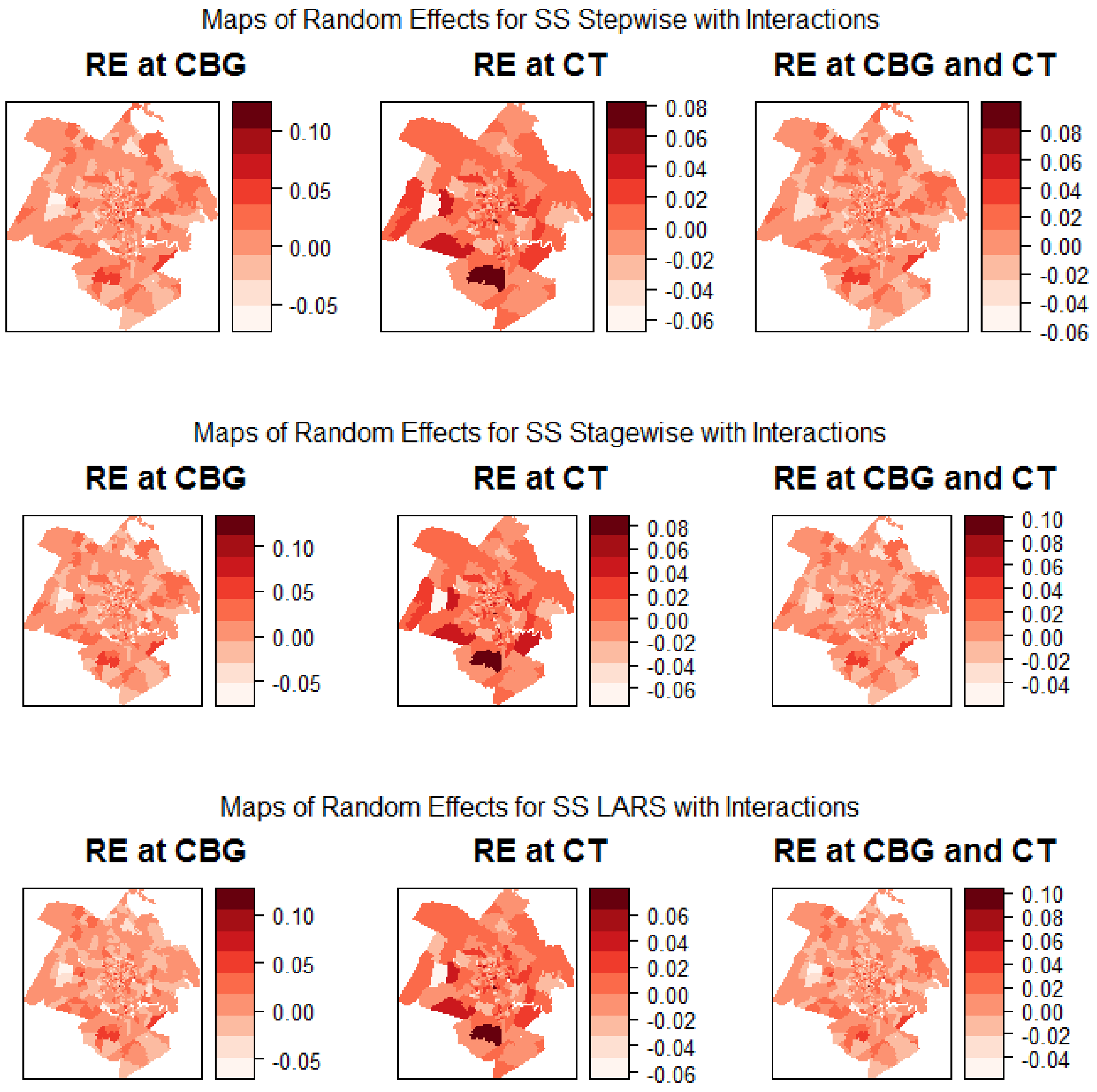

Figure S3. Maps of random effects at the census block group (CBG), census tract (CT), and CBG/CT for linear mixed-effects models of BMIZ using covariates selected by the spatial scale (SS) forward stepwise, forward stagewise, and LARS/lasso algorithms and including the covariate male and select interaction terms in the Richmond Metropolitan Statistical Area (MSA).
